# Image Restoration and Analysis of Influenza Virions Binding to Membrane Receptors Reveal Adhesion-Strengthening Kinetics

**DOI:** 10.1371/journal.pone.0163437

**Published:** 2016-10-03

**Authors:** Donald W. Lee, Hung-Lun Hsu, Kaitlyn B. Bacon, Susan Daniel

**Affiliations:** School of Chemical and Biomolecular Engineering, Cornell University, Ithaca, New York, United States of America; Emory University School of Medicine, UNITED STATES

## Abstract

With the development of single-particle tracking (SPT) microscopy and host membrane mimics called supported lipid bilayers (SLBs), stochastic virus-membrane binding interactions can be studied in depth while maintaining control over host receptor type and concentration. However, several experimental design challenges and quantitative image analysis limitations prevent the widespread use of this approach. One main challenge of SPT studies is the low signal-to-noise ratio of SPT videos, which is sometimes inevitable due to small particle sizes, low quantum yield of fluorescent dyes, and photobleaching. These situations could render current particle tracking software to yield biased binding kinetic data caused by intermittent tracking error. Hence, we developed an effective image restoration algorithm for SPT applications called STAWASP that reveals particles with a signal-to-noise ratio of 2.2 while preserving particle features. We tested our improvements to the SPT binding assay experiment and imaging procedures by monitoring X31 influenza virus binding to α2,3 sialic acid glycolipids. Our interests lie in how slight changes to the peripheral oligosaccharide structures can affect the binding rate and residence times of viruses. We were able to detect viruses binding weakly to a glycolipid called G_M3_, which was undetected via assays such as surface plasmon resonance. The binding rate was around 28 folds higher when the virus bound to a different glycolipid called G_D1a_, which has a sialic acid group extending further away from the bilayer surface than G_M3_. The improved imaging allowed us to obtain binding residence time distributions that reflect an adhesion-strengthening mechanism via multivalent bonds. We empirically fitted these distributions using a time-dependent unbinding rate parameter, *k*_*off*_, which diverges from standard treatment of *k*_*off*_ as a constant. We further explain how to convert these models to fit ensemble-averaged binding data obtained by assays such as surface plasmon resonance.

## Introduction

**Fig 1:**
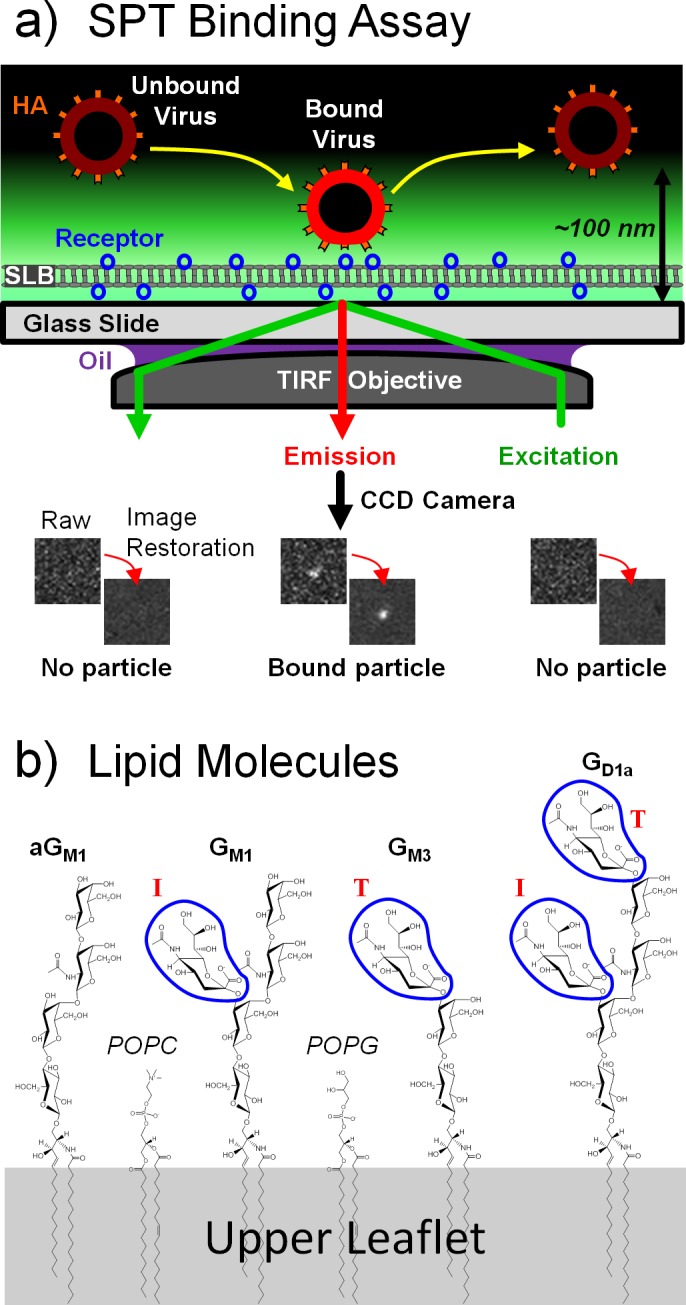
SPT binding assay and receptor structures. a) Setup of SLB on TIRF microscope. Evanescent field illuminates regions where SLB and virus interacts. b) Structure of the sialic acid receptors for X31 tested here. The α2,3-linked sialic acid groups are circled by the blue perimeters. Following the notation by Suzuki et al. [16], sialic acid groups are labeled as either being in the internal (I) or terminal (T) position.

Single-particle tracking (SPT) is a versatile technique for studying protein-protein binding interactions occurring at surfaces, particularly the binding of viruses to host cell membrane receptors [1–5]. Viral adhesion to host membranes is critical for viral infection, and dissecting this process is relevant for predicting virus emergence, determining susceptible hosts, or developing binding-inhibitory antiviral compounds. SPT often deploys the use of imaging techniques such as total internal reflection fluorescence (TIRF) microscopy, which can track fluorescent virions within a 100-nm distance away from a surface (Fig 1A). The viral receptor can be loaded onto a flat substrate by tethering receptors to polymers attached covalently to the substrate [6], adsorbing lipid vesicles containing the receptor lipid or protein [7], or forming supported lipid bilayers (SLBs) containing membrane receptors [4–5, 8–10]. The SLB option is advantageous because 1) the receptor type and surface density can be carefully controlled through bilayer preparation steps, 2) receptors are properly orientated in the membrane [11], 3) viral membrane fusion kinetics can be studied using the same assay [8, 12–15], and 4) mobile lipids allow the virus to recruit receptors and form multivalent bonds. However, the SPT-SLB assay contains several technical challenges with experimental design, image processing, and binding kinetic data analysis that limit its adaptation as a standard analytical tool. To increase the utility of SPT-SLB assays, we explain the cause of and demonstrate solutions to these issues as we study of influenza virus binding to several types of α2,3 sialic acid (SA) glycolipids.

One of the biggest barriers to using SPT for viral research is extracting particle information from images. Manually tracking particles is impractical, hence automated tracking software is needed and its proper performance is critical. Fluorescently labeled virions appear as white spots on a dark background, but often, dim particles exist that are only few pixels large and resemble bright shot noise. Dim particles are particularly detrimental to SPT binding studies because these are intermittently detected by the software and contribute many short, erroneous particle trajectories that skew the binding data. Increasing the particle fluorescence signal by using a more powerful excitation laser is not an effective solution to the problem because photobleaching destroys the signal before adequate data can be collected. Signal-to-noise ratios can be improved by developing better microscopy setups such as light sheet microscopy [17], using other dyes such as quantum dots [18–19], or using dye-free strategies [20], but not all setups and samples can accommodate these improvement. Hence, developing image-processing techniques provides a means to benefit a broader range of studies with minimal changes to experimental procedures.

Typical SPT image analysis software usually performs the following three steps: image restoration, particle detection, and particle trajectory linking. While much effort has been invested in improving particle detection [21–27] and particle linking algorithms [23, 27–33], less effort has been focused on developing image restoration algorithms for SPT application [34]. Particle tracking software generally underperforms when the signal-to-noise ratio (SNR) is below 4 [23]. Most SPT studies use image restoration techniques originally designed for standard photographs and videos [35–38], which do not adequately restore dim particles with features barely larger than that of shot noise pixels. To resolve the intermittent particle tracking errors, we developed and provide a SPT image restoration algorithm called STAWASP (Segmented Temporal Averaging While Avoiding Synced Pixels). This algorithm effectively uses both spatial and temporal information from the video pixels to stabilize particle intensities, preserve particle features, and remove substantial noise from videos.

Other challenges of SPT-SLB assays is customizing experiments for studying multivalent binding of viruses to receptor analogs, and interpreting the SPT binding/unbinding kinetic data. There are many experiment design choices to consider that affect the kinetic data, and we explain these choices in the context of designing experiments for studying influenza X31 virus (H3N2 strain), which is a model for other enveloped viruses. Enveloped viruses are often labeled with membrane fluorophores since they do not interfere with protein functions and can be used in membrane fusion/dye-dequenching studies [8, 13–15, 39–40]. Yet, quenched concentrations of membrane dye or inefficient labeling can generate substantial numbers of dim particles that are intermittently observable. Hence, tracking membrane-labeled X31 virus serves as a useful test case for developing our image restoration algorithm and data analysis strategies.

A typical influenza virion surface contains roughly 400–500 hemagglutinin (HA) proteins [41] that govern the multivalent binding of the virus to sialic acid receptors on host cells. In this work, we measured the binding times of influenza viruses to SLBs that separately contained glycolipids aG_M1_, G_M1_, G_M3_, or G_D1a_ (Fig 1B). Except for aG_M1_, all glycolipids contain the α2,3 SA linkage but with different peripheral oligosaccharide structures. These structures serve to impart differences in binding behavior that can be resolved when tracking stochastic binding events instead of ensemble-averaged binding events. We are also interested in counting the binding attempts made by viruses, which is not observable when using traditional binding assays that measure the change in collective adsorbed mass of viruses on a receptor-coated surface.

Generally, viruses bind better to SA at the terminal position [16, 42–43], but SPR studies have shown that X31 virus does not bind to G_M3_ even though it also has a terminal SA [7]. Contrary to this study, X31 virus can infect chicken red blood cells that have been incubated with G_M3_ receptors [44]. We show that the flu virus does bind weakly to G_M3_, observable with the experiment conditions and image restoration used in this work. Additionally, we show that binding residence time distributions reflect the adhesion-strengthening process via multivalent bonds that enable viruses to bind stably to host membranes [45–48], and we present strategies to characterize these kinetics.

## Materials and Methods

### Producing Lipid Vesicles for SLBs

Supported lipid bilayers (SLBs) containing glycolipids were made from lipid vesicles. To make the lipid vesicles, 1-palmitoyl-2-oleoyl-sn-glycero-3-phosphocholine (POPC) and 1-palmitoyl-2-oleoyl-*sn*-glycero-3-phospho-(1'-*rac*-glycerol) (POPG) [Avanti Polar Lipids, Alabaster, AL] were separately dissolved in chloroform, while glycolipids monosialotetrahexosylganglioside (G_M1_) [Avanti Polar Lipids, Alabaster, AL], monosialodihexosylganglioside (G_M3_), disialoganglioside (G_D1a_), and asialoganglioside (aG_M1_) [Sigma-Aldrich, St. Louis, MO] were separately dissolved in a 2:1 chloroform to methanol solution. Glycolipids and POPC solutions were mixed together to yield 1 mol % of glycolipids, while POPG was added when necessary to maintain the same anionic charge density across all lipid samples (equivalent to 2 mol % of monovalent anionic lipids). Lipids were dried under vacuum for 3 hours and rehydrated in MES buffer (1 mM MES, 150 mM NaCl, pH 7) such that the final lipid concentration was 1 mg/mL. Lipids were extruded 10 times using a 1-directional, 20-mL lipid extruder [Northern Lipids Inc., Burnaby, Canada] and a 50 nm pore-size polycarbonate membrane [GE Healthcare, Pittsburgh, PA] to yield 100 nm-diameter vesicles as determined by dynamic light scattering [Malvern Instruments, Worcestershire, UK]. Extruded lipids were collected in a new vial to ensure all lipids have passed the filter.

### Measuring Surface Charges of Vesicles and Viruses

To ensure long-range electrostatic forces do not influence binding, the surface charges of the vesicles and lipid vesicles were measured using a zeta potential analyzer [Malvern Instruments, Worcestershire, UK]. Lipid vesicles at 0.25 mg/mL dilutions or X31 virus (dye-labeled and unlabeled) at 0.008 mg/mL dilutions were added to zetacells (model DTS 1061) and subjected to light scattering measurements under an electric field. Triplicate measurements were performed at pH 7, 150 mM NaCl.

### Assembling the Microfluidic Device

Virus binding experiments were performed inside a microfluidic device as shown elsewhere [15]. Glass coverslip slides (No. 1.5 thickness) [VWR, Radner, PA] were cleaned for 10 min using a piranha solution composed of 30 vol % hydrogen peroxide solution (contains 50 wt % H_2_O_2_) [Sigma-Aldrich, St. Louis, MO] and 70 vol% concentrated sulfuric acid [VWR, Radner, PA]. Glass slides were stored in deionized water until use. A polydimethylsiloxane (PDMS) [Dow Corning, Midland, MI] mold of microfluidic channels (135 μm wide, 75 μm high, and 1.5 cm long) was prepared on top of a patterned, hydrophobic silica wafer produced at the Cornell Nanofabrication Facility (CNF). An air-dried glass slide and the PDMS mold were annealed together after a 30-second oxygen plasma cleaning step at 700 μmHg oxygen pressure. Tygon tubes (0.02” ID x 0.06” OD) [Saint-Gobain Performance Plastic, Worcester, MA] were attached to the microfluidic device such that one end is submerged into the loading solution and the other end is attached to a syringe pump [Harvard Apparatus, Holliston, MA]. The microfluidic device was setup on an inverted total internal reflection fluorescence (TIRF) microscope with a 100x oil immersion objective and 1.46 numerical aperture [Carl Zeiss, Oberkochen, Germany. Model Axio Observer Z1]. A 561 nm laser was shined at a 70° incidence angle to generate an evanescent wave that illuminates the virus within ~100 nm from the glass-water interface. The camera in the microscope is a Hamamatsu ImageEM C9100-13 (Hamamatsu Photonics, Bridgewater, NJ).

### Forming SLBs Inside Microfluidic Channels

SLBs that act as host membrane mimics were formed by rupturing lipid vesicle on hydrophilic glass surfaces [9, 49]. Lipid vesicle solutions at 1 mg/mL vesicle concentration were loaded into the microfluidic channels at a flow rate of 100 μL/min for 1 min. Vesicles were incubated in the channels for 4 hours to form high quality, high-quality bilayers. Excess vesicles were rinsed away by flowing MES buffer at a flow rate of 100 μL/min for 3 min.

### Storing X31 Viruses Until Use

X31 (H3N2/Aichi/68) influenza were obtained from Charles River [Charles River Laboratories, North Franklin, CT] at a protein concentration of 2 mg/mL, which comes in a frozen state. The stock virus solution was defrosted, aliquoted into 5 μL volumes and stored at -80°C until used. An aliquot of virus was freshly thawed and used for each experiment. While this procedure requires viruses to undergo two thaw cycles that decreases viral infectivity relative to fresh virus, it is repeatable and out-competes the other option to store the virus liquid state until each use. The drop in infectivity for a 2^nd^ thaw cycle has been reported to be from 10^8.6^ to 10^7.0^, whereas degradation over time at 0°C liquid state causes a larger drop in infectivity to 10^6.5^ [50]. The spherical morphology of virus was fairly well preserved after a 2^nd^ thaw cycle, as confirmed in a separate EM study using the same virus batch [51].

### Labeling the Viral Membrane with R18

To label the viral envelope with lipophilic fluorescent dye, 5 μL of the virus solution, 250 μL of MES buffer, and 4 μL of 0.01 mg/mL ethanol-dissolved octadecyl rhodamine B (R18) [Invitrogen, Carlsbad, CA] were mixed together in a vial. The mixed solution was gently sonicated in a water bath for 30 min at 25°C in the dark. Unincorporated R18 dye was filtered out using a G-25 sephadex spin column [GE Healthcare, Pittsburgh, PA] at 3000 RPM (743 RCF), and the eluted virus solution was stored in a LoBind vial [Eppendorf, Hamburg, Germany] to prevent loss of viral particles to vial surfaces while conducting the experiments. Before use, 250 μL of filtered virus solution was diluted with 1 mL of MES buffer. Note the labeling of virus with membrane dyes has already been shown to not affect HA function [52–53].

### Determining the Virus Concentration and Size

The stock X31 virus solution at an initial viral protein content of 2 mg/mL was diluted to 0.016 mg/mL. The virus solution was loaded into a flow chamber used in the NanoSight system (Malvern Instrument, Worcestershire, UK). Videos of particles floating in the solution were analyzed using the NanoSight software to extract the virus concentration.

### Setting the Camera Rate and Experiment Time

SPT studies face an unusual sensitivity to the image capture rate of SPT microscopy [1]. Sensitivity is attributed to the fact that a continuous time data of binding events, which could last anywhere from 0 to infinite time, are being sampled via a camera taking images at discrete time intervals with a set exposure time. Similar issues are explained by the Shannon Nyquist Sampling Theorem [54] and Bally et al. [1]. Short term binding events will inevitably be lost during the dead time between images, which means the overall binding event data will be affected by the camera setting. Increasing the imaging rate (by shortening the dead time) is not always viable as this would lead to excessive dye photobleaching issues and loss of data about long-term binding viruses. The camera setting was thus set according to the minimum binding residence time resolution desired relative to a reference time scale. We set the reference time scale based on that of virus-mediated *de novo* clathrin mediated endocytosis (CME), which takes roughly 3 min [55]. To encompass the CME timescale, camera was set to take images at 1 s intervals using a 100 ms exposure time for a total duration of 20 min (*t*_*movie*_ = 20 min).

### Conducting SPT Binding Experiments

Labeled viruses were loaded into a SLB-coated microfluidic channel at a flow rate of 100 μL/min for 1 min. The flow was stopped by balancing the tube inlet and outlet pressures, which takes at most 1 minute to equilibrate. Stagnant flow conditions are necessary to prevent shear forces from affecting the binding/unbinding kinetics, especially of concern for weak binding interactions. Videos were recorded 1 min after the virus was introduced and the flow was stopped. The excitation laser was turned off in-between images during the 900 ms dead time to prevent excessive photobleaching of fluorophores while recording the video. After experiments, a 20 vol % bleach solution was sent into the microfluidic channels to inactivate viruses. All experiments were performed at 25°C.

### Restoring Images and Tracking Particles in SPT Videos

Shot noise from SPT videos was removed using our image restoration algorithm called STAWASP, to stand for Segmented Temporal Averaging While Avoiding Synced Pixels. Details about STAWASP are provided below. Particles were detected using a custom algorithm that looks for circular regions that are brighter than the background noise intensities (Part D in S1 Text). Particles’ trajectories were determined using a basic algorithm that links particles from adjacent video frames together that are within 3 pixels away from each other (Part E in S1 Text). Any remaining and obvious errors in trajectories were corrected manually to increase the overall data accuracy, though the number of manually corrected trajectories constitutes a small portion of all trajectories returned by the automated tracking algorithm. All algorithms were developed using MATLAB (Mathworks, Natick, MA).

### Defining a “Binding Event” via Visual Cues

Unlike SPR/QCM where the adsorbed mass to a surface can be detected, or AFM where binding force can be measured, SPT relies on visual cues that a binding event has occurred. These visual cues are not always obvious as virions can undergo stop-and-go motions. There are two criteria that can be used: Criteria 1 –a visible virus is always considered bound, or Criteria 2 –a visible virus that is immobile for at least a minimum duration time (*t*_*cutoff*_) is considered bound. With Criteria 1, a mobile virus is treated as only 1 binding event regardless if this particle stops at and moves to several places. The concern with Criteria 1 is that the more interesting “stopping” events are ignored and the virus may not always be in contact with the SLB while moving. For instance, the virus could be rolling along the SLBs, unbinding and rebinding to receptors, or simply floating near the field of view of TIRF without making contact. With Criteria 2, a single mobile virus can generate multiple binding events when it remains temporarily immobilized during the stop-and-go motion.

While Criteria 1 may be more appropriate only when studying the lateral diffusion of viruses into coated pits [56], we used Criteria 2 because an immobile virus is most certainly bound to the SLB. In this regime we are then corresponding most closely to the biological situation of virus binding events that lead to *de novo* CME occurring at stationary sites [57]. However, we must decide a minimum binding time for a binding event (*t*_*cutoff*_), otherwise there would be no visual cue to discern a bound virus from a floating virus. We chose a *t*_*cutoff*_ of 5 frames (or 5 s) based on the performance of the automated particle tracking software (Part F in S1 Text). Note that the choice to *t*_*cutoff*_ will always be arbitrary because we are relying on visual cues, and not physical contact, to discern binding event. Ideally, we would want to capture events upon contact with the smallest *t*_*cutoff*_ possible. Furthermore, the camera exposure time and frame rate sets a minimum cutoff time. We later investigate how the choice of *t*_*cutoff*_ affects the binding data.

### Filtering Biased Binding Events

When analyzing the binding residence time distributions, some binding events must be discarded due to ambiguity or bias. Particles that existed since the first frame (left-censored data) of the video were discarded since the actual binding time is uncertain. Those that bound after half of the movie time (½ *t*_*movie*_) were also discarded because binding events that last longer than ½ *t*_*movie*_ cannot be observed in a fair manner as those that last for shorter times. Virions that stayed bound by the end of the movie (right-censored data) were included in the data if they initially bound before ½ *t*_*movie*_. Due to the filtering of biased binding events, binding survival curves are drawn only up to ½ *t*_*movie*_.

## Results and Discussion

### Establishing Conditions for High-Quality SLBs

**Fig 2:**
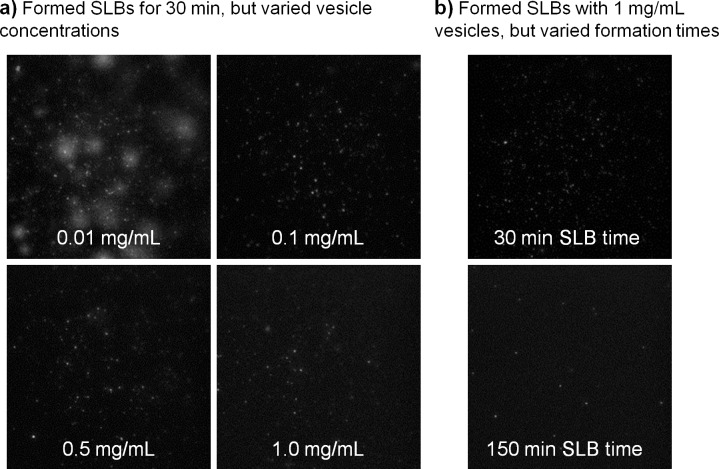
Optimizing SLB formation to reduce nonspecific binding. a) SLBs were formed over 30 min using 1% aGM1 receptor-less lipid vesicles at varying concentrations (white text). X31 nonspecifically bound and spontaneously fused to SLBs (as noted by the radial release and diffusion of R18 into the supported bilayer) formed with low vesicle concentrations. b) The SLB formation time (white text) was varied while maintaining a constant 1 mg/mL vesicle concentration. Nonspecific binding was reduced greatly when SLBs are formed over at least 150 min. All images in panel a and b, were taken 30 min after loading the X31 virus.

There are several ways to form SLBs: Langmuir Blodgett, lipid film rehydration, or vesicle rupture [9]. When using microfluidics, the vesicle rupture strategy is an effective means to form SLBs as it simply requires loading solutions of lipid vesicles into the channels. However, such method has previously been reported to cause low-quality SLBs if the devices are plasma cleaned [58]. To overcome this issue, which can be especially problematic when studying weak binding interactions, we determined conditions at which high-quality SLBs form with very minimal defects that viruses bind nonspecifically to by optimizing vesicle concentration and SLB formation time. Some issues with using lower vesicle concentrations are the creation of defective SLBs that induce nonspecific binding and spontaneous virus fusion (Fig 2A), most likely due to interaction with edges of SLB patches. Defective SLBs like these could be useful for developing antiviral surfaces and may be worth exploring as a future work. However, since the focus of this work is on studying binding interactions, we sought conditions for reducing nonspecific binding events. Using at least 1 mg/ml vesicle concentration worked well. The SLB formation time is also important and can vary based on pH or salt [59], lipid compositions [60], and glass surface treatment [61]. For our SLBs, at least 3 hours were needed to reduce non-specific binding of viruses to negligible levels (Fig 2B). Additional blocking steps were not required since SLBs themselves act as passivation layers. Dye-labeled bovine serum albumin, which is a typical blocking agent, did not bind to these bilayers to any detectable level (data not shown). Furthermore, inclusion of blocking agents could instill uncertainty, as one must confirm that these do not coat the receptors, coat the viruses, or disrupt the SLB.

### Controlling for Long-Range Nonspecific Electrostatic Binding

Controlling long-range, electrostatic interactions is essential for ensuring that the observed binding events are due to specific virus-receptor interactions. The net charge varies across aG_M1_ (0 charge), G_M1_ (-1 charge), G_M3_ (-1 charge), and G_D1a_ (-2 charge). To maintain the same receptor concentration and charge density of the bilayers, charged lipids must be added. Negatively-charged POPG lipids were added to equalize the negative charge density across all of the SLBs (Table 1). Positively charged lipids could also be used to neutralize charges from the lipids, but since viruses are negatively charged, clusters of positively charged lipids could potentially induce non-specific binding. The surface charge of the virus must also be monitored, as the usage of too much lipophilic dye R18, which is positively charged, could change the polarity of the virus surface. The zeta potentials of all lipid vesicles and dye-labeled X31 virus were negative and less than 5 mV in magnitude (Table 1). These magnitudes are much lower than 30 mV zetapotentials that could lead to nonspecific electrostatic attraction/repulsion between the virus and receptors [62]. The high salt content of the buffer also helps screen the charges. Long-range, nonspecific interactions are unlikely to play a major role in the viral binding kinetics here, enabling us to focus more on measuring the binding of viruses to their putative receptors.

**Table 1:** Zeta potential measurements of vesicles and viruses. suv = small unilamellar vesicles.

Sample	Zeta potential (mV)
1% aGM1 2% POPG 97% POPC suv	-3.45±0.50
1% GM1 1% POPG 98% POPC suv	-3.32±0.59
1% GM3 1% POPG 98% POPC suv	-4.60±1.19
1% GD1a 0% POPG 99% POPC suv	-3.47±0.73
X-31 virus with R18	-2.02±0.26
X-31 virus without R18	-10.11±0.55

### Introducing STAWASP Image Restoration Algorithm for SPT

Shot noise is rooted in low-signal, digital images due to the discrete photon collection method of digital cameras and complex electronic signal amplification hardware. In SPT videos, the airy rings of dim particles are barely larger than 2x2 pixels, making them nearly indistinguishable from noise. There are three major image restoration approaches: spatial, temporal, and spatio-temporal filtering. Spatial filtering removes static noise pixels based on how abnormal a pixel’s intensity is compared to neighboring pixels. This method could produce artifacts such as particle blurring. Temporal filtering evaluates how pixel intensity changes over time to remove high-frequency noise fluctuation. However, it can cause particles to become blurred or faded. Spatio-temporal filtering combines aspects from both approaches, but this could become computationally expensive if it requires tracking local spatial regions over time.

**Fig 3:**
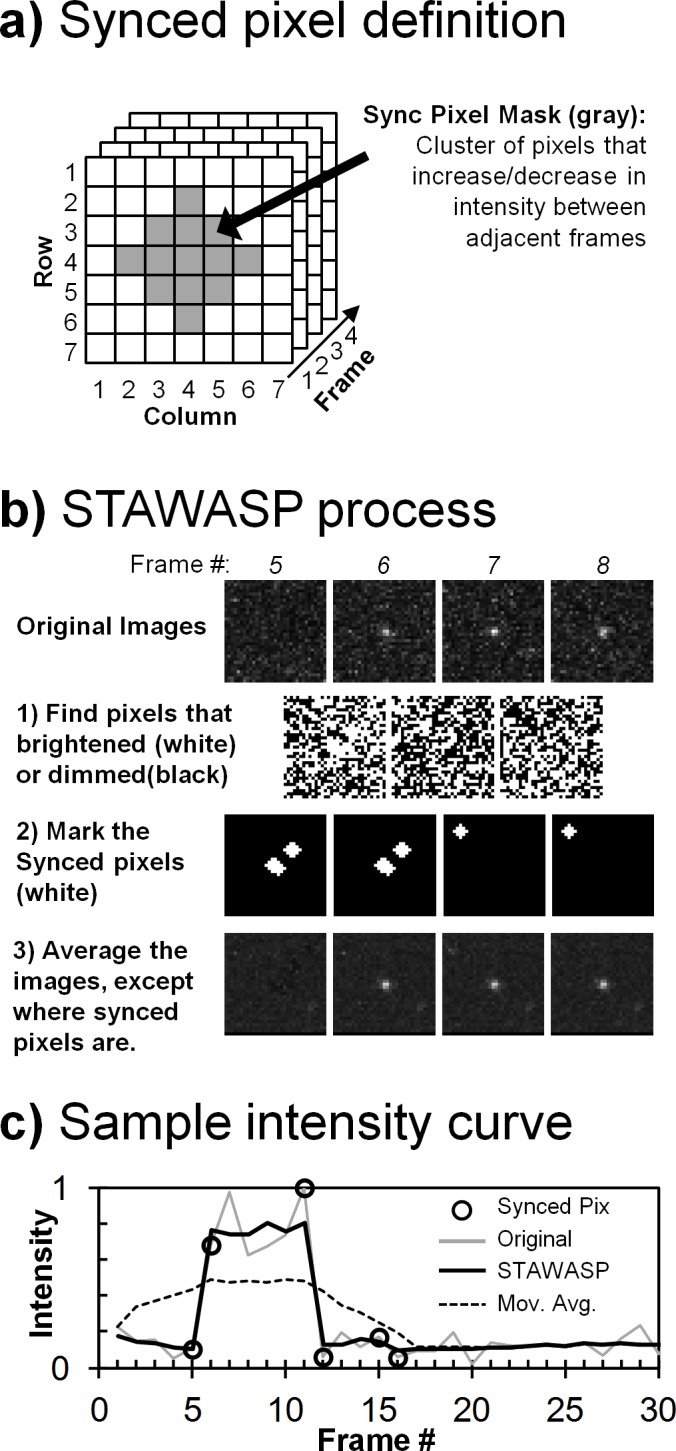
STAWASP algorithm. a) A local cluster of pixels (gray pixels) must either increase or decrease in intensity between consecutive frames to be classified as synced pixels. The pixel cluster size must be larger than a pixel and smaller than the particle of interest. Here, at least 80% of the gray pixels must be synced. b) The steps to determine synced pixels are portrayed here for a particle appearing at frame 6. Since synced pixels are determined using two adjacent frames, synced pixels are marked on both of those frames. Note that false synced pixels can be generated by random noise fluctuations, but these do not necessarily cause a false particle to appear. c) The intensity trace for the center pixel of the particle images part B is shown when using STAWASP or the regular 10-frame temporal averaging scheme. With STAWASP, temporal averaging is performed in a segmented fashion such that temporally adjacent, synced pixels are not averaged together.

Our STAWASP image restoration method can be classified as a spatio-temporal filter, and the algorithm is explained in Fig 3. In short, this algorithm removes noise and preserves particle signals by averaging images (or stacking images) together in spatially and temporally-divided pixel segments. The unique feature of this algorithm is the method at which segments are determined with little input about what is or is not a particle. Segments are determined based on how synchronous a cluster of pixels fluctuates with time. For instance, the chance all 13 pixels in a circular area (Fig 3A) synchronously increase intensity from one frame to the next is improbable due to random noise and highly probably if a particle appears/disappears/moves. Pixels that change intensity synchronously are marked as “synced pixels” (Fig 3B). Each pixel in the 2D image is then averaged through time, but the averaging is done in segments separated by the appearance of synced pixels (Fig 3C). We provide the STAWASP software as a supplemental MATLAB stand-alone and source code files, and the logic and usage of STAWASP are explained in detail in Parts A and B in S1 Text.

### Comparing the Performance of STAWASP

**Fig 4:**
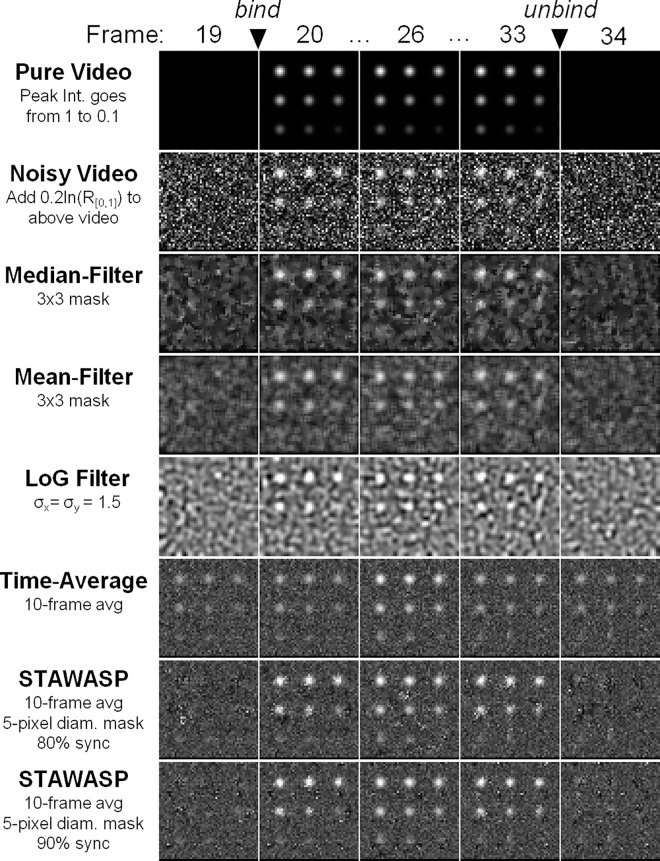
Testing various image restorations on a simulated movie. A simulated movie with noise was generated to compare image restoration performance (see Supporting Materials 1.3 for simulation details). The pure video shows particles with varying intensities appearing at frame 20 and disappearing after frame 33. Noise is added according to the function N = -0.2ln(R), where R is a uniform random number from 0 to 1. The SNRs of the 9 particles, from top left to bottom right are as follows: 5.0, 4.4, 3.9, 3.3, 2.8, 2.2, 1.6, 1.1, 0.5. The LoG (Laplacian of Gaussian) spatial filtering method is described by others [34, 63].

The performance of the STAWASP image restoration was compared against other common methods, using a first simulated, noise-ridden video of binding particles (Fig 4). Simulation details are provided in Part C in S1 Text. The signal-to-noise ratios (SNRs, defined here as the particle peak intensity divided by the standard deviation of the noise intensity) were varied between 0.5 to 5. The Mean, Median, and LoG (Laplacian of Gaussian) noise filters intermittently revealed particles with SNRs ≥ 2.2, whereas particles with SNRs < 2.2 were undetectable. The 10-frame temporal averaging method was able to reveal particles with SNRs ≥ 1.1, but all the particles faded in and out of view. The STAWASP algorithm was able to reveal particles with SNRs ≥ 1.1, and it preserved the appearance/disappearance times of particles with SNRs ≥ 2.2.

No image restoration is perfect, and a common artifact of the STAWASP algorithm are short-lived bright pixels throughout the movie, which is a result of the inability to distinguish a small cluster of bright noise from an actual particle. These artifacts are dealt with later by the particle detection (Part D in S1 Text) and tracking algorithms that filter out false particles (or shot noise that looks like particles) based on the criteria for a real binding event, as discussed above. We use a custom tracking algorithm that links particles together within 3 pixels between frames (Part E in S1 Text). Since determining the improvement of tracking in real SPT videos is difficult due to the inability to know the “true” binding events, we instead used a simulated video of particles and noise to assess the overall improvement in tracking (Part F in S1 Text). The particle detection and tracking results with and without STAWASP are provided in Part F in S1 Text. In short, STAWASP-restored videos enabled our SPT software to extract binding residence time curves that converges to the true data for when *t*_*cutoff*_ > 5 s, whereas the true data is not obtainable without any restoration. Note that for actual SPT videos involving X31 viruses, we manually correct any obvious, erroneous trajectories to increase the accuracy of the data.

### Applying STAWASP for Real X31 Virus Binding Videos

**Fig 5:**
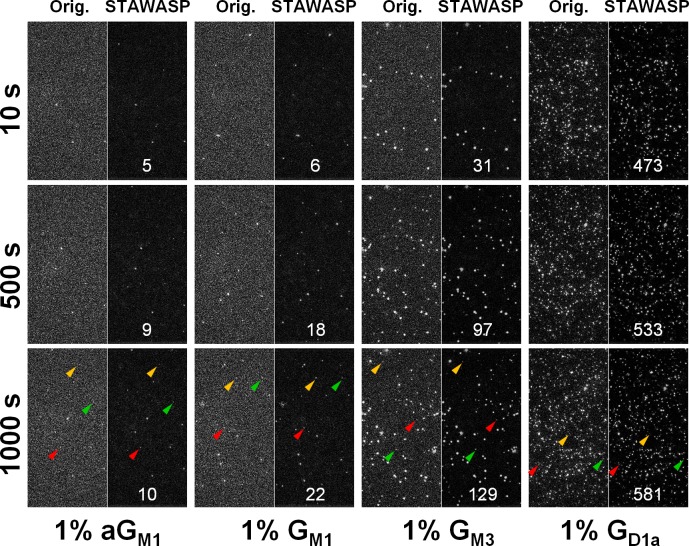
Sample images of viruses binding to glycolipids with and without image restoration. Few examples of restored particles are shown by the colored triangles. The white number shows the particle count, *P*_*count*_, for the right half of each image, which represents a physical size of 82 μm high x 41 μm wide using 512 x 256 pixels. Note that *P*_*count*_ is not the same as *N*. We show *P*_*count*_ for qualitative comparisons only, since quantitative comparisons must be done using *N* instead, which is determined after the particle linking step. The time on left is the video recording time, which starts ~60 s after the virus is loaded, and therefore some virus exists at time 0. We show images starting at 10 s merely because the performance of STAWASP is optimal after 10 frames. We provide original movies without any image restoration for the first 300 s as S1–S4 Videos, played at 10x real time where 1 frame = 1 second. STAWASP-enhanced movies are provided for X31 binding to G_M3_ (S5 Video) and G_D1a_ (S6 Video). Variations in particle intensities are caused by several factors, such as variable levels of dye that incorporated into the viral membrane, different degrees of photobleaching, and uneven microscope illumination (viruses in the center are generally brighter than those near the edges). [Image Processing Note: All images, including left and right halves, have undergone background subtraction as explained in Part D in S1 Text, and intensities were linearly scaled. See S1–S4 Videos for original images].

We next show real images of viral binding before and after using STAWASP image restoration (Fig 5). The clarity provided by the restored image is critical for obtaining accurate number statistics of viral binding. A qualitative assessment of the virus binding microscopy images shows that X31 binds most frequently to G_D1a_, less frequently to G_M3_, and negligibly to G_M1_. The control case shows a minimum level of nonspecific binding of X31 to aG_M1_ SLBs, which confirms high-quality SLBs were formed inside microfluidic channels. The binding levels of virus to aG_M1_ bilayer also serves to characterize nonspecific binding levels, which can be contributed either by microdefects in the SLBs or denatured HA proteins that can insert hydrophobic residues into the SLBs.

Some interesting observations from the SPT videos are surface-diffusing viruses. As mentioned in the Methods section, surface-diffusing viruses can be a result of rapid binding/unbinding events, tethered virus to mobile receptors, or rolling viruses. There are higher levels of surface-diffusing viruses in G_D1a_ bilayers compared to G_M3_ bilayers. The most likely explanation seems to be that the “diffusion” is actually rebinding events happening in rapid succession. For G_D1a_, a virus rebinding to the bilayer is a highly probable event due to favorable binding interactions. For G_M3_, the rebinding probability is much lower, and thus virus particles are more likely to float away from the field of view rather than bind to an adjacent site. The other possibilities, either that the virus is rolling or moving with a few attached glycolipids, seem less likely because the virus is much larger than a lipid and the G_D1a_ receptor density is too high. There are roughly 420 receptors per pixel (assuming a lipid occupies 0.61 nm^2^ [64] and 1 pixel = 25600 nm^2^), hence it would difficult for a virus to traverse long distances without forming immobilizing, multivalent bonds.

### Analyzing the Binding Frequency Rate and Rate Parameter *k*_*on*_

Before discussing the virus binding kinetic data, we must first clearly define variables associated with SPT data analysis, as summarized in Table 2.

**Table 2:** Variables involved with SPT data analysis.

Variable	Definition
*P*_*count*_	Number of particles detected in an image
*N*	Number of binding events
*N*_*+*_	Accumulated number of binding events since the movie started
*N*_*-*_	Accumulated number of unbinding events since the movie started
*t*	Time elasped in the movie
*t*_*res*_	Binding residence time (or contact time) of virus to receptors
*t*_*cutoff*_	Minimum binding residence time required for a binding event
*t*_*movie*_	Total duration time of the SPT video

Note that *N* ≤ *P*_*count*_ (unless *t*_*cutoff*_ = 1 frame) because not all particles seen in the movie satisfy the binding event criteria that a particle must remain immobile for longer than a certain cutoff time. Furthermore, when discussing SPT data, distinguishing normal time *t* and residence time *t*_*res*_ is important. For instance, *N* vs *t* plots portray the net number of binding events as a function of time, whereas *N* vs *t*_*res*_ plots portrays how many binding events last longer than *t*_*res*_ time (which is a survival function).

**Fig 6:**
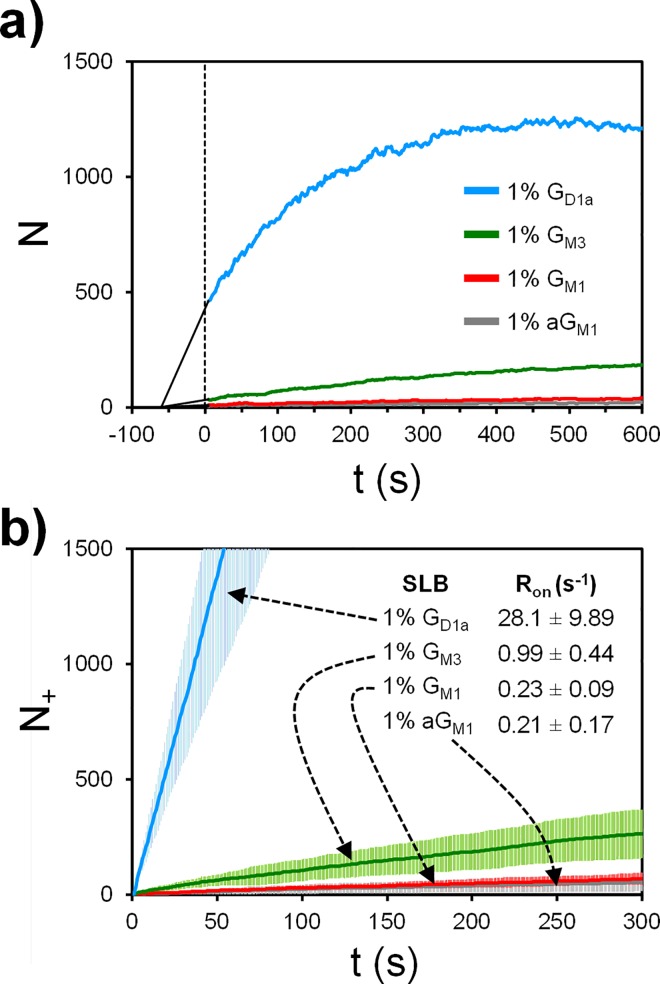
Two types of binding rate data of X31 to various receptors. **A**) N vs t plots for one representative data set from a triplicate set. Negative time means time before recording, and the solid black lines are just an extrapolation to -60 s when the viruses are estimated to first reach the SLBs. B) N_+_ vs t plots, averaged over a triplicate set of data. The slope of this plot corresponds to binding frequency rate R_on_. The error bars are the standard deviations, computed using 3 different trials.

We first analyzed the *N* vs *t* plots for X31 binding to the SA receptors. *N* is related to the other variables by the equation N(t) = N_+_(t)–N_-_(t) + N_0_, where N_0_ is the N when the movie starts. The N(t) data clearly shows that G_D1a_ bilayers have a higher capacity to hold onto viruses (Fig 6A, blue line), versus other receptors. One interpretation of the slowly rising net virus binding curve of G_M3_ (Fig 6A, green line), relative to G_D1a_’s curve, is to say G_M3_ is not a functional receptor. In fact, SPR studies concluded that G_M3_ is not a receptor for X31 [7], though the authors studied viruses binding to lipid vesicles instead of SLBs, using half the G_M3_ concentration than what was used here, and while applying a slight hydrodynamic flow that could prevent weak binding events. However, having a dN/dt ~ 0 does not mean there is no binding rate, similar to how when a system reaches equilibrium, a forward/reverse reaction still exists.

The *N*_*+*_ vs *t* plots (Fig 6B) show that X31 does in fact bind more frequently to G_M3_ than to aG_M1_ and G_M1_. The slope of the *N*_*+*_ vs *t* plots yields the binding frequency rate *R*_*on*_, and *R*_*on*_ for G_M3_ is 3 STDs higher than that of aG_M1_ and G_M1_ cases. Our data suggest that G_M3_ can be a functional receptor for the virus.

**Fig 7:**
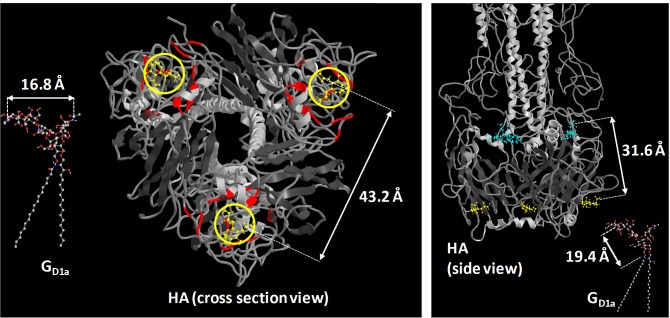
Using structural arguments to understand binding results. Left) View of HA protein head group in relation to G_D1a_. Red regions show the binding pockets and yellow circles show where sialic acid are located. Right) Side view of HA protein in relation to G_D1a_. Teal molecules are sialic acids at potential secondary binding sites. The SLB would be on the bottom side of the protein, while the viral membrane would be on the top side. The hemagglutinin structure and sialic acid positions were obtained by Sauter et al. [65] PDB ID: 1HGG.

Despite G_D1a_ and G_M3_ each having a terminal SA known to promote binding [16], X31 binds to G_D1a_ ~30 fold more frequently than to G_M3_. This cannot be explained by the presence of 2 SA per G_D1a_ molecule, as spatial distances between SA do not allow them to bind to 2 binding sites of an HA trimer (Fig 7), including alternative binding sites located at the HA1/HA2 junction [65]. Additionally, the lack of binding of X31 to G_M1_’s internal SA suggests that the virus is not binding to the same internal SA in G_D1a_. The extended distance of terminal SA from the SLB hydrophobic layer appears to promote binding. Access to G_M3_’s internal SA may be sterically hindered by the close proximity of terminal SA to the bilayer.

Binding rates are often characterized via a binding rate constant *k*_*on*_, but SPT data do not provide a single value for *k*_*on*_ as we will discuss the reasons for later. Starting with the standard approach for finding a single *k*_*on*_ value, the *k*_*on*_ parameter can be solved for via the relation *R*_*on*_ = *k*_*on*_[V][SA]*A*_*cam*_ where *A*_*cam*_ is 6274 μm^2^ for our camera field of view, [V] is the visible virus concentration in the bulk solution, and [SA] is the surface density of free receptors in the SLB. Since *R*_*on*_ is determined using early time points of the experiment, this enables the assumptions that [SA] and [V] are approximately equal to initial values right when the virus is loaded into the channels. Therefore we can treat [SA] ≈ [*SA*]_init_ and [*V*] ≈ [*V*]_init_ to estimate the binding rate constant *k*_*on*_. The concentration [*V*]_init_ is ~4.5 pM based on Nanosight measurements, and the receptor surface density [*SA*]_init_ is ~16500 μm^-2^ for 1 mol % assuming a lipid occupies 0.65 nm^2^ [64]. With these constants, the values for *k*_*on*_ are (4.4±3.6)x10^2^, (5.5±2.0)x10^2^, (2.1±0.9)x10^3^, and (6.0±2.1)x10^4^ 1/Ms for aG_M1_, G_M1_, G_M3_, and G_D1a_ respectively, when using the binding event criterion *t*_*cutoff*_ = 5s. The *k*_*on*_ values for G_M3_ and G_D1a_ are within the expected range found by other binding assays, which have reported *k*_*on*_ = 2x10^3^ M^-1^s^-1^ for multiple HAs binding to fetuin [66], *k*_*on*_ = 3.61x10^4^ M^-1^s^-1^ for soluble HA binding to G_D1a_ [67], and *k*_*on*_ = 1.6x10^6^ M^-1^s^-1^ for X31 virion binding to vesicles containing 0.5 mol% Neu5Acα2-3nLc4Cer [7].

**Fig 8:**
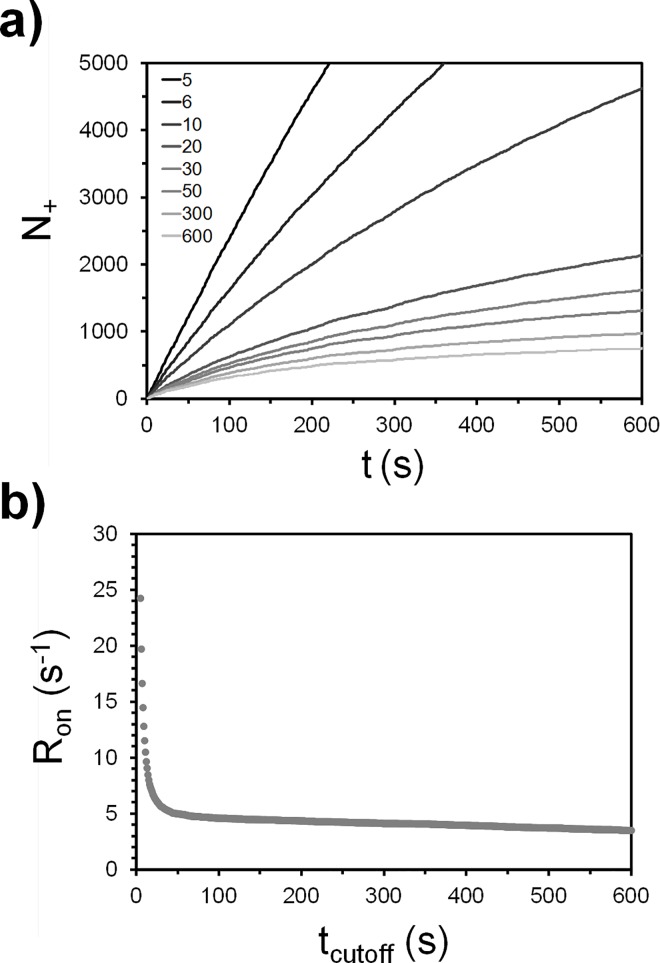
Observing how binding kinetics changes with choice of *t*_*cutoff*_ for a 1% G_D1a_ trial. a) *N*_*+*_ vs *t* at varying *t*_*cutoff*_ settings shown in the legend. b) *R*_*on*_ vs *t*_*cutoff*_ plot showing how *R*_*on*_ is affected with *t*_*cutoff*_ choice.

We now discuss why there can be multiple values of *k*_*on*_ and caution against the direct comparison of *k*_*on*_ from SPT assays to ensemble assays. Both *k*_*on*_ (or *R*_*on*_) depend on the choice of *t*_*cutoff*_ because *t*_*cutoff*_ dictates how many binding events are included in a data set (Fig 8A). For instance, by setting *t*_*cutoff*_ to be infinite, no binding events will exist that meets this requirement, hence, *k*_*on*_ → 0. Conversely, setting *t*_*cutoff*_ to be 0 would make *k*_*on*_ seem to diverge to infinite because there will be no distinction between a binding event, elastic collision, or floating virus that is simply visible on the camera. Since the chance for a binding event to exceed a certain *t*_*cutoff*_ value is dictated by the binding residence time distribution, this means a relationship between *k*_*on*_ and *k*_*off*_ exists and the two parameters are not entirely decoupled as one would normally expect. In other words, the ability to observe a binding event is affected by the ability of the virus to stay bound long enough to be observed. To understand this relationship, we generated a plot for SPT data, that relates *R*_*on*_ (which is directly proportional to *k*_*on*_) to the choice for *t*_*cutoff*_ (Fig 8B).This plot also serves to facilitate the comparison of binding data taken across various *t*_*cutoff*_ settings.

### Analyzing the Binding Residence Time Distribution and Unbinding Parameter *k*_*off*_

**Fig 9:**
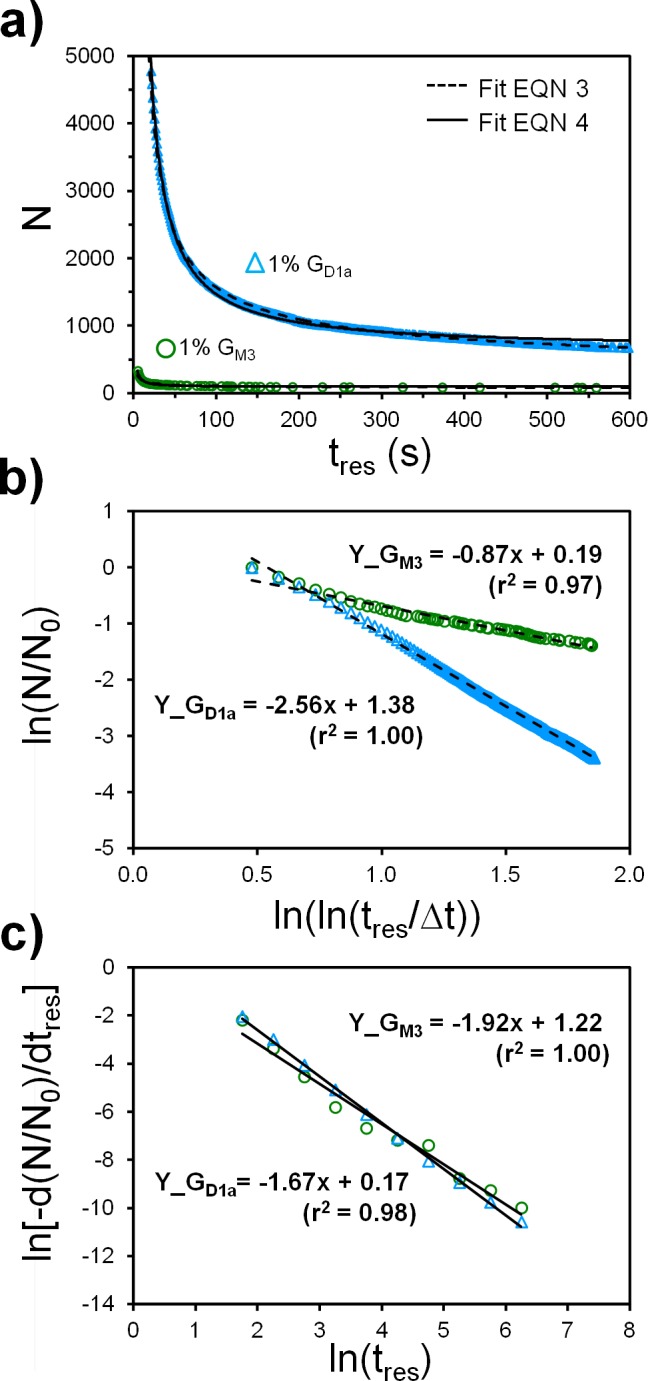
Representative X31 binding survival curves and empirical fits. a) The number of virus bound, N, is plotted against the residence time, *t*_*res*_, to yield a survival curve for binding. The Eq 3 fit parameter for G_M3_ is [A = 0.87, B = 0.19] and G_D1a_ is [A = 2.56, B = 1.38]. The Eq 4 fit parameters for G_M3_ is [A = 2.14, B = 1.63] and G_D1a_ is [A = 2.06, B = 1.72]. Note that the Eq 4 fit parameters differ from those found from the related log plots in panel c. The binning of the binding data in the log plots causes an approximation error. b) Log plots used to derive Eqn 3. c) Log plots used to derive Eqn 4 as used Bally et al.[1] (the data has been binned).

We next analyze the *N* vs *t*_*res*_ plots that portray information about the binding residence time distribution and unbinding rates (Fig 9A). The unbinding curves did not agree with the 1:1 binding model (Eq 1), and double exponential fit model (Eq 2).

Eq 1: single binding energy population

$$N=N_{0}\exp(-k_{\mathrm{off}}t_{\mathrm{res}})$$

Eq 2: double binding energy populations

$$N=N_{0,A}\exp(-k_{\mathrm{off},A}t_{\mathrm{res}})+N_{0,B}\exp(-k_{\mathrm{off},B}t_{\mathrm{res}})$$

Rather, N decays in an unusual logarithmic fashion with respect to *t*_*res*_, as also noticed by norovirus binding kinetics studies [1–2] and our recent work with parvovirus binding to transferrin receptors [48]. Alternative analytical models are generally lacking for multivalent binding systems and recent studies have begun to address this issue [1–2, 5, 48, 68]. Here, we searched for an empirical model to describe the overall unbinding probability of the virus by testing various log plots between *N* and *t*_*res*_ until a linear relationship was found (Fig 9B and 9C). Two possible fit equations for *N* vs *t*_*res*_ are provided (Eqs 3 and 4). Note that Eq 4 mimics the approach used by Bally et al. [1], while Eq 3 is developed by us and also describes the binding residence distribution of parvovirus binding to transferrin receptors [48].

Eq 3: empirical fit derived from Fig 9B

$N=N_{0}\exp(B){\left(\ln\frac{t_{\mathrm{res}}}{\Delta t}\right)}^{-A}$ where Δt is a reference time interval ≤ *t*_*cutoff*_. We used Δt = 1s based on the time interval between images. Note that all equations here apply only when *t*_*res*_ ≥ *t*_*cutoff*_.

Eq 4: empirical fit derived from Fig 9C and proposed by Bally et al [1]

$$N=N_{0}\left[1-\frac{\exp(B)}{1-A}\left(t_{\mathrm{res}}^{1-A}-t_{0}^{1-A}\right)\right]\text{if}\;\text{A}\neq 1$$

$$N=N_{0}\left[1-\exp(B)\ln\frac{t_{\mathrm{res}}}{t_{0}}\right]\text{if A}=1$$

Unbinding rates are often characterized via an unbinding constant *k*_*off*_, which in turn is related to binding force. Since Eqs 1 and 2 failed to fit our data, this suggests that the unbinding kinetics does not follow a constant *k*_off_ value, complicating matters. We thus assumed *k*_*off*_ is not a constant and could vary with *t*_*res*_. To extract *k*_*off*_, we equated the empirical fits with the 1^st^ order dissociation equation (d*N*/d*t* = -*k*_*off*_*t*) and solved for *k*_*off*_. The resulting equations for *k*_*off*_ are shown as Eqs 5 or 6 (derivations are in Part G in S1 Text).

Eq 5: *k*_*off*_*(t*_*res*_*)* based on Eq 3

$$k_{\mathrm{off}}(t_{\mathrm{res}})=\frac{A}{t_{\mathrm{res}}\ln\left(\frac{t_{\mathrm{res}}}{\Delta t}\right)}$$

Eq 6: *k*_*off*_*(t*_*res*_*)* based on Eq 4

$$k_{\mathrm{off}}(t_{\mathrm{res}})=\frac{\exp(B)t_{\mathrm{res}}^{-A}}{1-\frac{\exp(B)}{1-A}\left(t_{\mathrm{res}}^{1-A}-t_{0}^{1-A}\right)}\text{if A}\neq 1$$

$$k_{\mathrm{off}}(t_{\mathrm{res}})=\frac{\exp(B)t_{\mathrm{res}}^{-1}}{1-\exp(B)\ln\frac{t_{\mathrm{res}}}{t_{0}}}\text{if A}=1$$

Since *k*_*off*_ is a function of *t*_*res*_, this would imply that the binding force changes depending on the contact time between the virus and receptor. This is in good agreement with recent work by H. Witt and coworkers showing that binding force of multivalent bonds does change with contact time [69]. For viruses, the general term for increasing binding force over time is adhesion-strengthening [45–47], which includes co-receptor binding, conformational changes of viral proteins, and multivalent binding due to receptor diffusion within the target bilayer. Since the HA proteins do not significantly change conformation upon binding [65] or under the neutral pH conditions used here, adhesion strengthening is thus most likely caused by multivalent binding of the virus to the tightly-packed, mobile SA receptors on the SLB. In support of this, the estimated number of glycolipids per contact area is ~90, assuming a virus diameter is 150 nm, a lipid molecule occupies 0.61 nm^2^ of the SLB [64], and the contact area is roughly 30% of the virus cross-sectional area based on cryo-EM pictures of virus-membrane contacts [70]. Furthermore, the glycolipid mobility was confirmed by fluorescence recovery after photobleaching (FRAP) experiments (see [71–72] and Part H in S1 Text).

A competing interpretation of our unbinding curves is that *k*_*off*_ spans a wide range of values due to a wide distribution of virus sizes and thus degree of multivalent binding. To make an empirical model under this interpretation, one would sum many exponential functions for 1:1 binding model that are multiplied by a weight function that reflection the virus size distribution. This approach would also assume multivalent bonds effectively act as a single bond with a stronger binding force. However, the virus distribution is narrow and morphology is uniform according to particle size and EM studies [51]. Additionally, AFM studies showed complex unbinding process [73] that would be inconsistent with the idea that multivalent bonds can be treated as a single bond. Overall, the interpretation that *k*_*off*_ varies with *t*_*res*_ because of adhesion-strengthening via multivalent binding is most likely to agree with the biology involved in this study. The remaining question is why does *k*_*off*_ approach infinity if we allow *t*_*res*_ to approach 0? Theoretically, this is because as *t*_*res*_ goes to 0, an elastic collision between virus and membrane will count as a binding event, and thus *k*_*off*_ will be substantially high to reflect a lack of binding force.

### Bridging SPT and SPR Data Analysis

Since protein-ligand binding interactions are often studied via ensemble assays such as surface plasmon resonance (SPR) and quartz crystal microbalance (QCM), we explain how to fit ensemble data using our empirical model from SPT data. Ensemble assays collect data on the net adsorbed mass on a surface over time, which would be similar to the *N* vs *t* data from our SPT assay. However, unlike SPT assays, ensemble assays require two experimental procedures to decouple binding and unbinding kinetics, noted as the “association” and “dissociation” phases. The virus must be loaded during the association phase to observe both binding and unbinding, and then a virus-free buffer is loaded during the dissociation phase to observe mainly unbinding. The procedure often requires the virus to be subjected to a gentle hydrodynamic flow that could shear off very weakly bound viruses and affect the final data. Also, rebinding events that occur during that dissociation phase are difficult to completely filter out and could lead to disagreements between expected and actual unbinding curves [74].

**Fig 10:**
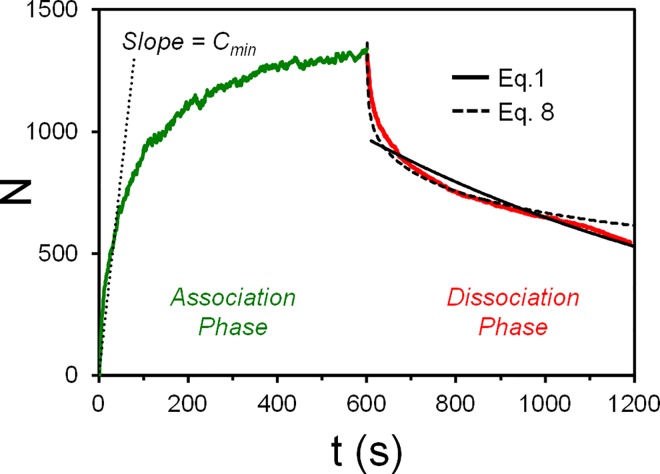
SPR-like curve assemble generated from SPT data for the purpose of comparing and contrasting the approaches. The fit parameters for Eq 8 are A = 2.0, C = 47 (which is greater than C_min_ = 16.4). The fit parameters for Eq 1 are N_0_ = 971 and *k*_*off*_ = 0.001 s^-1^).

With SPT, we can extract association and dissociation curves using stagnant conditions and without conducting a separate dissociation phase procedure, simply by filtering binding events that occur during the “dissociation phase” that is set at the image processing stage. To make SPR-like curves, we plot *N* vs *t*, but with few differences. *N* is now set to start at 0 at *t* = 0 by removing binding events that occurred at or before the first frame, and a dissociation phase session is defined to start after time *t*_*diss*_ = ½ *t*_*movie*_, in which new binding events that occurred after that are ignored (Fig 10).

The unbinding curve can be fitted using a modified form of Eq 3, which the residence time *t*_*res*_ is converted into normal time *t* as shown in Eq 7. Eq 7 simply states that number of binding events remaining at *t* > *t*_*diss*_ is the sum of the binding events that occurred at *t* < *t*_*diss*_ and lasted until time *t*.

Eq 7

$$N_{diss}(t)=\sum_{t'=0}^{t'=t_{diss}-\Delta t}\Delta tR_{on}(t')\exp(B){\left[\ln\frac{t-t'}{\Delta t}\right]}^{-A}\text{for}\;t\geq t_{diss}$$

Since ensemble assays do not yield *R*_*on*_(*t*) directly, an simplifying assumption must be made. We approximate Δ*tR*_*on*_(*t*) as a constant, C, based on the *N*_*+*_ vs *t* plots showing a fairly linear relation and constant slope *R*_*on*_ (Fig 6) This yields the fit Eq 8, which would be used if one were given SPR-like data without knowing exactly what *R*_*on*_(*t*) is.

Eq 8

$$N_{diss}(t)=\sum_{t'=0}^{t'=t_{diss}-\Delta t}C{\left[\ln\frac{t-t'}{\Delta t}\right]}^{-A}\text{for}\;t\geq t_{diss}$$

*C* is the number of binding events that occur on average within a time increment Δt. One could instead use a time-varying *C* fit parameter, *C*(*t*), but it may lead to over-fitting issues. A lower bound value of *C* can be set based on the initial slope of the *N* vs *t* curve during the association phase, denoted as *C*_*min*_. Optimal fit parameters can be determined via an iterative search strategy. An example of how our adhesion-strengthening model (Eq 8) performs against the standard 1:1 binding model (Eq 1) is shown in Fig 10. The exponential fit model failed to fit the steep drop in unbinding immediately after the dissociation phase start, whereas our empirical model fitted main features of the curve.

## Conclusions

We assessed of the SPT binding assay platform, image restoration, and data analysis for use in virus-membrane binding studies. We developed an image restoration algorithm called STAWASP to enhance dim particle signal and improve SPT binding data quality. STAWASP restored particles that had a SNR as low as 1.1, and preserved appearance/disappearance time of particles with an SNR as low as 2.2. The image restoration enabled the SPT software to extract accurate binding residence curves when tracking particles lasting at least 5 frames. However, a small number of trajectories must still be manually corrected. Fully automated tracking without any error is currently not possible and is an active research area for machine-learning algorithms.

We explained data analysis strategies for X31 binding to various glycolipids, and showed X31 weakly binds to G_M3_ and to G_D1a_ ~28 times more frequently than to G_M3_, despite both containing terminal sialic acid that should promote receptor binding [16]. On the other hand, ensemble assays and cell infectivity assays had mixed results about whether or not the virus can bind to G_M3_ [7, 44]. The accessibility of SA appears highly restricted by the close proximity of it to the SLB lipid core.

The binding residence distribution curves show an interesting time-dependent *k*_*off*_, which is unusual since *k*_*off*_ is often assumed to be a constant. The empirical model agrees with the interpretation that viruses increase their binding force to host membrane with longer contact time via an adhesion-strengthening process. For X31 virus, adhesion-strengthening most likely occurs via multivalent bonds that form when mobile receptors in the SLBs are recruited to the virus, as opposed to any protein conformation changes. We provide a general strategy to apply our empirical model to fit ensemble assay unbinding data (Eq 8) that feature a sharp decline in the dissociation curve, followed by a slow decay.

The combined image restoration and analysis tools developed here is easily extendible to other studies that involve imaging low-fluorescence particles binding stochastically with surfaces. We have recently applied these tools for tracking parvovirus binding to transferrin receptors [48], in which both were labeled with limited fluorophores and were difficult to observe without image restoration. Another example where this approach could be useful is for studying viruses that undergo membrane fusion upon receptor binding (such as parainfluenza). In such case, viruses are usually labeled with a quenched membrane dye and would be difficult to track until membrane fusion and dye-dequenching occurs.

## Supporting Information

S1 File MATLAB files for STAWASP and particle detection. (ZIP)

S2 File Excel file containing raw data for X31 binding to SLBs. (XLSX)

S1 Text Supporting Information document. (PDF)

S1 Video X31 binding to 1% aG_M1_ bilayers, original. (AVI)

S2 Video X31 binding to 1% G_M1_ bilayers, original. (AVI)

S3 Video X31 binding to 1% G_M3_ bilayers, original. (AVI)

S4 Video X31 binding to 1% G_D1a_ bilayers, original. (AVI)

S5 Video X31 binding to 1% G_M3_ bilayers, restored with STAWASP. (AVI)

S6 Video X31 binding to 1% G_D1a_ bilayers, restored with STAWASP. (AVI)
