# Supplementary figures and images for: Image Restoration and Analysis of Influenza Virions Binding to Membrane Receptors Reveal Adhesion-Strengthening Kinetics
Source: PLoS One. 2016 Oct 3;11(10):e0163437. doi: 10.1371/journal.pone.0163437 (PMC5047597; doi:10.1371/journal.pone.0163437)

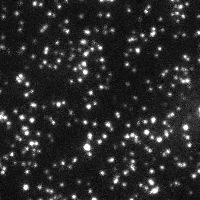

Supplement: S1 File — (ZIP) [file pone.0163437.s001.zip › Code for simulation and detection/Demo_X31 Detection.png]

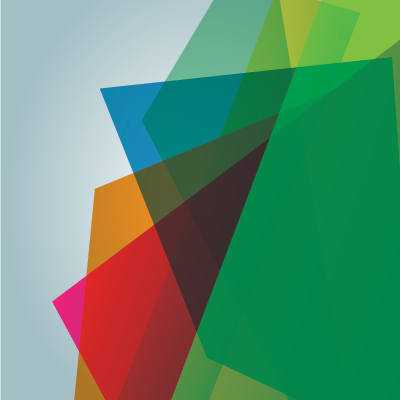

Supplement: S1 File — (ZIP) [file pone.0163437.s001.zip › Option 1 - STAWASP EXE/splash.png]
