# Supplementary material for: Image Restoration and Analysis of Influenza Virions Binding to Membrane Receptors Reveal Adhesion-Strengthening Kinetics
Source: PLoS One. 2016 Oct 3;11(10):e0163437. doi: 10.1371/journal.pone.0163437 (PMC5047597; doi:10.1371/journal.pone.0163437)
Supplement: S1 Text — (PDF) [file pone.0163437.s003.pdf]

S1 Text. Supporting Information.

Image restoration and analysis of influenza virions binding to membrane receptors reveal adhesion-strengthening kinetics

Donald W. Lee, Hung-Lun Hsu, Kaitlyn B. Bacon, and Susan Daniel
School of Chemical and Biomolecular Engineering, Cornell University, Ithaca, New York, United States of America

Overview

This document is divided into a software-related section and an experiment-related section. The software-related section provides explanation of the image restoration and particle tracking algorithms. The experiment-related section provides results of extra control experiments and derivations of fit equations for the binding residence time distributions.

SOFTWARE-RELATED

Part A. Logic behind STAWASP image restoration. **2**

Part B. STAWASP GUI usage. **3**

Part C. Generation of simulated videos. **5**

Part D. Particle detection. **7**

Part E. Particle linking. **9**

Part F. Testing particle detection and linking performance. **10**

EXPERIMENT-RELATED

Part G. Derivation of fit equations. **12**

Part H. Confirming lipid mobility using Fluorescence Recovery after Photobleaching (FRAP). **14**

Part A. Logic behind STAWASP image restoration.

Here we explain the mechanism behind STAWASP noise removal process. We first define the intensity of a pixel (*I*) as the summation of the signal (*S*) and noise (*N*).

$$I(r,c,t) = S(r,c,t) + N(r,c,t)$$

where *r* is the row location, *c* is the column location, and *t* is the time or frame number of the pixel. We can break up the noise into spatial and temporal components.

$$I(r,c,t) = S(r,c,t) + N_m(r,c,t) + N_t(t)$$

where, N_m = mean noise that varies only in space (N_m is a positive number), and N_t = fluctuation intensity around the mean noise (N_t can be negative or positive).

The spatial noise can be removed using a background subtraction whereas the temporal noise can be removed using a temporal averaging scheme. As the time span of averaging goes to infinity, $N_t(t)$ approaches 0. However, this works only when there is no particle signal. If there is a particle signal, then temporal averaging must be done carefully in segments. The STAWASP algorithm creates these segments based on where and when synced pixels are found. Ideally, synced pixels should only be found when particles appear, disappear, or move. However, noise can generate synced pixels by coincidence. We can estimate the percentage of falsely synced pixels using a binomial distribution.

$$P_{sync} = \frac{Q!}{M!(Q-M)!} p^M (1-p)^{N-M}$$

P_{sync} : probability a cluster of pixels are in sync due to random shot noise

Q : Number of pixels in the neighbor mask for determining a synced cluster

M : Number of pixels within the neighbor mask that actually must be in sync

p : Probability intensity increases or decreases between adjacent frames ($p \approx 0.50$)

Our default setting generates a 5-pixel diameter circular neighbor mask consisting of 15 pixels (Q = 13), in which 80% of the pixels must be synced (M = 10). The probability random noise causes synced pixels is roughly 0.0349. The lower the P_{sync} value the stronger the noise reduction scheme. However, Q and M are capped by the smallest size of the particles' airy rings in the videos. To reduce the number of falsely synced pixels, one could impose more stringent rules about what to consider as a pixel intensity change. For example, a rule can be imposed specifying that the intensity of a pixel must change by at least a minimum value between adjacent frames to be counted as an increase or decrease in intensity for the STAWASP algorithm.

Part B. STAWASP GUI usage.

Installation:

Option 1) The standalone STAWASP.exe file requires the MATLAB Runtime Compiler to be installed prior to running the stawasGUI.exe. The MRC installer can be found in the MathWorks website, and the required version is for MATLAB R2015a (8.5).

Option 2) The stawasp GUI can be installed using the STAWASP_WebInstall.exe file instead, which will automatically download the necessary MRC file required to run the program.

Option 3) If MATLAB is installed, the source code can be used directly. Save the source code files into a folder. Open MATLAB, set the folder path to the saved folder, and then enter "stawaspGUI" in the command line. Note that the Image Processing Toolbox is required.

Basic Usage:

1) Open STAWASP.exe for the stand-alone version or run stawaspGUI.m in the MATLAB console.

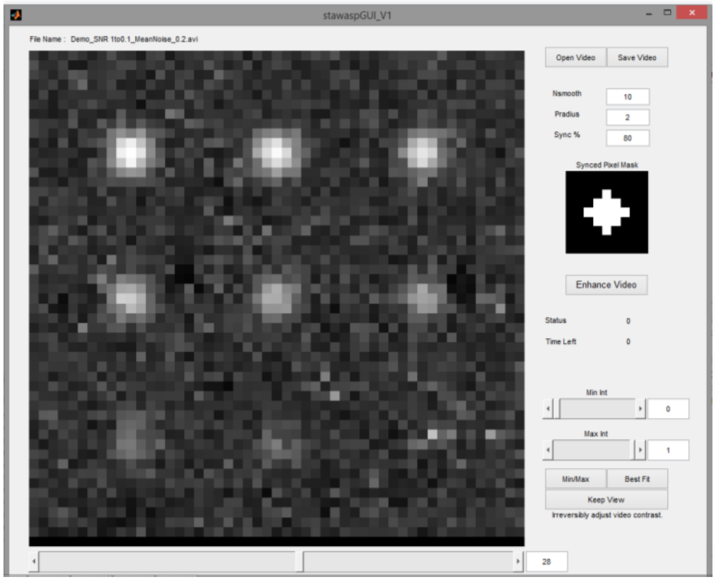

Fig S1 Graphical User Interface for STAWASP Image Restoration.

2) Open the video to be processed. Note: limited video file types are supported, and we recommend processing videos with the uncompressed AVI format.
3) Configure the STAWASP algorithm parameters.
   - **Nsmooth**: Maximum number of frames that can be averaged together. Generally, a larger Nsmooth will get rid of noise better.
   - **Pradius**: The radius, in pixels, of the neighborhood mask. Ideally, this will be slightly larger than a pixel, and smaller than the particle of interest. EX: If particles have radius of 3, Pradius should be set between 1 and 2.
   - **SyncPerc**: The percentage of the pixels inside the neighborhood mask that actually need to be synced. Generally, a higher SyncPerc will remove noise better.

4) Once the parameters are set, click “Enhance Video” to begin the STAWASP image restoration process. Note that this can take a few minutes depending on the video size and computer. A video of size 512x512 pixels and 1200 frames takes 250 sec to process with a single core CPU 1.8 GHz and 6 GB ram.
5) Optional: Adjust the image brightness to the desired setting and push “Keep View” to make the intensity changes permanent.
6) Save the video. The output will be an uncompressed AVI file.

Part C. Generation of simulated videos.

Simulated videos are helpful for testing the performance of image restoration and particle tracking software. We highlight the main steps and codes used to generate these videos.

Step 1: Generate particle information [Code: GenerateParticles.m]

The particles' locations, intensities, and appearance/disappearance times were created using random number generators or manually, depending on the type of video to be generated. Particle data was stored in MATLAB structure variable called "tracker". Inside the tracker structure is a field called "History", which contains a 5-column matrix storing the following data per particle: Col 1) video frame numbers in which the particle exists, Col 2) the particle image pixel row locations, Col 3) the particle image pixel column location, Col 4) particle intensity, and Col 5) particle area.

Step 2: Create a video without noise [Code: CreatePureVideo.m]

Particles were drawn onto the blank video (which is an HxWxZ matrix) according to the particle data stored in the "tracker" variable. We made the particle intensities follow a Gaussian shape with a sigma width of 1.5 pixels around each particle's centroid location.

Step 3: Add shot noise to the simulated video [Code: CreateNoiseVideo.m]

Noise was added to the video made in Step 2. We first studied the background noise of real SPT videos and then chose an appropriate noise model. The background noise is consistent with shot noise that follows a Poisson distribution (Fig S2).

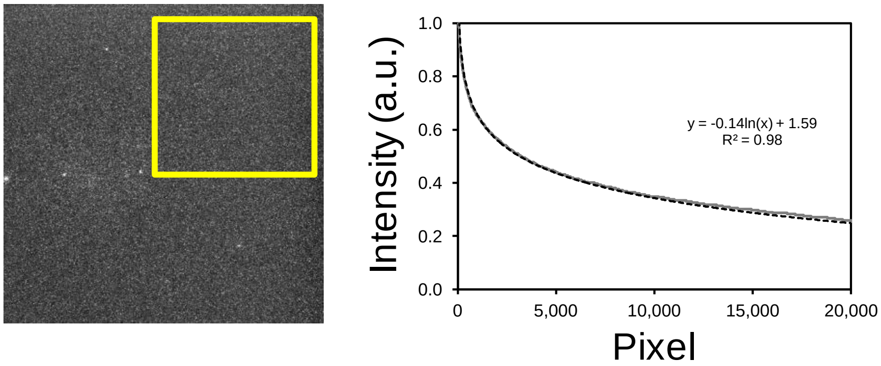

Fig S2   Background noise intensity distribution from real SPT video. The pixel intensities in the image background area (yellow box) were sorted and plotted as an intensity distribution, which reflects that of shot noise. Therefore we used the logarithmic model to mimic shot noise in simulated videos next.

Noise intensities were added to simulated videos using on the following equation: Noise = -MeanNoise*log(R), where R is a uniform random number from 0 to 1and MeanNoise is a user-set constant number between 0 and 1. Noise was added to every pixel in the video, and pixels with an intensity greater than 1 were capped at an intensity of 1 (Fig S3).

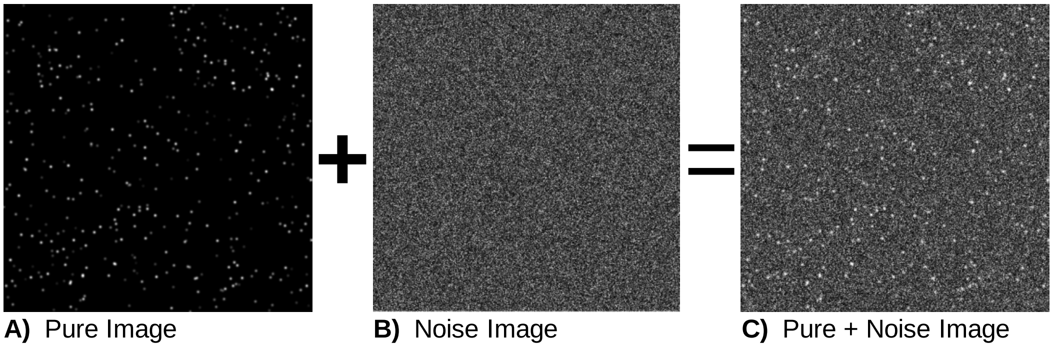

Fig S3 Generating movie of particle binding. A) Image of just the particles (without noise) was created using particle data. B) A pure noise image was generated separately. C) The noise was added to the pure image to create the final image for testing purposes.

Part D. Particle detection.

There are many particle detection algorithms in existence to choose from. Since we deal with particles in high densities, we avoided using Gaussian fitting methods for particle detection that are computationally expensive. To detect particles, we find regions that lack local intensity minima clusters, which normally correspond to a particle region. A particle is then found by searching for the local intensity maxima that reside in these regions lacking a high cluster of local intensity minima. In the last step, particles that do not fit the user-defined intensity and particle size threshold values are removed. The step-by-step particle detection process is portrayed in Fig S4-5, and it is implemented by the file code named ParticleFilter_V5.m.

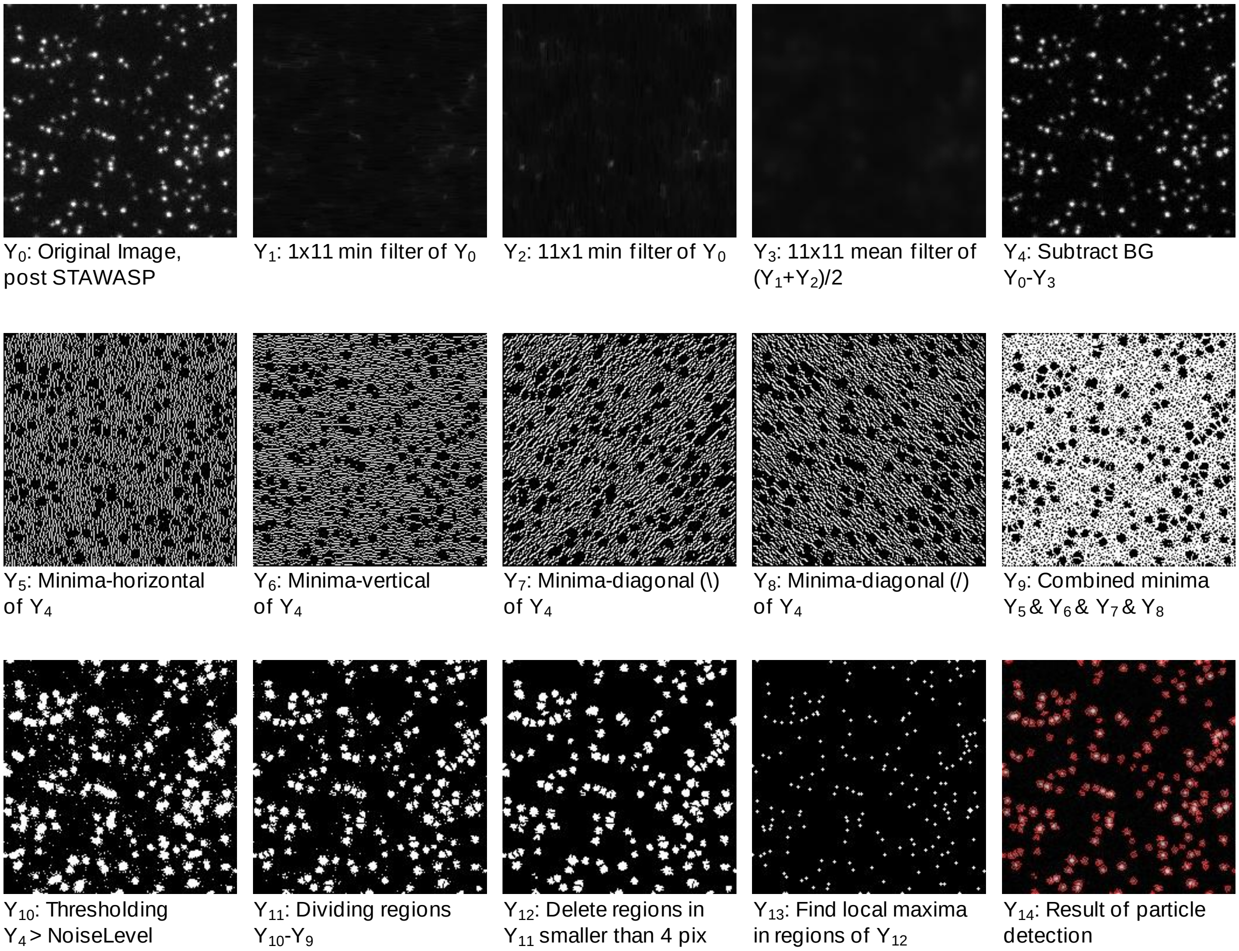

Fig S4 Flow chart of particle detection process. The “Minima” treatment finds the local minimal amongst 3 adjacent pixels in the direction specified after the dash. Note that for Y_{12}, the area of the particle that will be excluded can be adjusted by the user (here, it is set to 4).

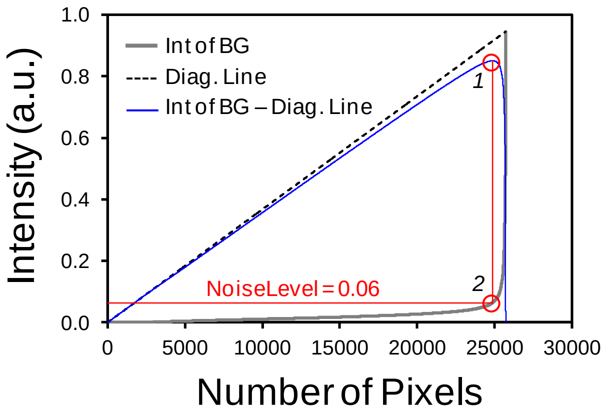

Fig S5 Automatic determination of Noise Level based on the intensity values of Y_4 that overlaps Y_9. Pixel intensities are first sorted and then are plotted (Int of BG). A diagonal line is drawn next (Diag.. Line). The two lines are then subtracted to yield the difference (Int of BG – Diag. Line), and the maximum point of the line is used to determine the NoiseLevel.

Part E. Particle linking.

After finding the location of the particles in each frame, each particle's trajectory must be linked through time. For this task, we use a simple nearest-distance particle linking algorithm due to the rapid speed and computational simplicity. Particles that are roughly in the same place (within a cutoff radius or 3 pixels = 480 nm) in adjacent images are tracked as a single particle. If a particle in one frame is not found in the next frame, then this particle's trajectory is ended. The particle trajectories can sometimes be ended prematurely due to the failed detection of the particle. This is usually caused by extreme shot noise or blinking particles. Intermittent detection error of stationary particles generates multiple trajectories that overlap the same spot but at different times. We automatically link these trajectories into one because the probability that 3 or more trajectories overlapping the same spot is rare, unless it is the same stationary particle that was intermittently detected. Furthermore, the binding kinetics is more tolerable to accepting 1 long incorrect binding event as opposed to many short and incorrect binding events. In the final step, any remaining errors in tracking results are manually corrected.

Part F. Testing particle detection and linking performance.

In order to confirm that the image enhancement, particle detection, and tracking algorithm are working properly, we calibrated the software on a simulated video containing 1000 particles of known locations and unbinding/binding times. The binding residence time distribution of all events was set to follow a single exponential decay function $N = N_0 exp(-0.10 F)$, where F is the image frame number (1 frame = 1 sec). The particle intensity was set to 0.44 and noise with a mean intensity and standard deviation of 0.2 was added to the video. The final video has a particle signal-to-noise ratio of 2.2. The particle detection method described above was used to detect particles before and after image restoration. We chose to focus on STAWASP and the 10-frame moving average method due to their similar temporal filtering processes. Particle detection results show how image restoration affects the particle detection performance (Fig S6).

Compared to detecting particles without any image restoration, the STAWASP-restored video reduced the false particles to 41% and missed particles to 5% while the moving-average-restored movie reduced the false particles to 45% and missed particles to 19%. False particles are difficult to remove using only particle detection algorithms since false particles caused by bright noise pixels look fairly similar to particles. Fortunately, false particles do not persist for long durations and can be removed during the particle linking step by setting a minimum tracking time duration.

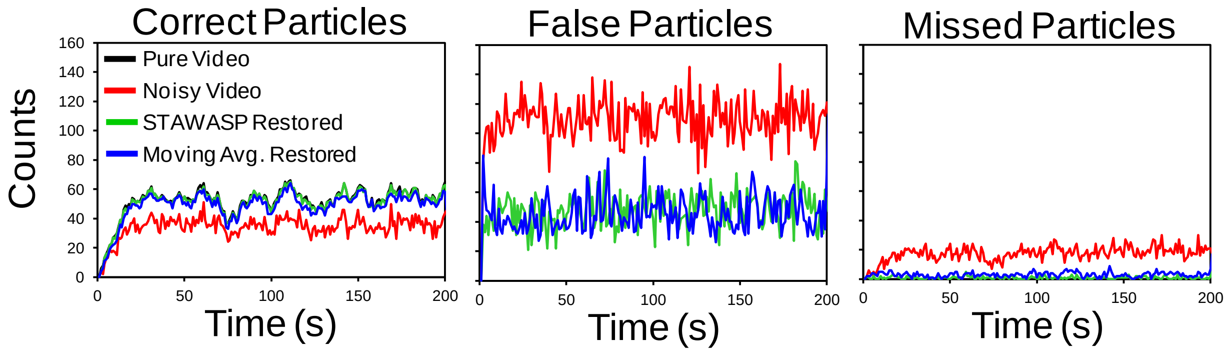

Fig S6 Particle detection results of the simulated particle binding video before or after image restoration. Settings for the STAWASP-restored video are Nsmooth = 10, Pradius = 2, and SyncPerc = 80%. The Moving Avg-restored video uses a 10-frame moving average. The particle detection was set to filter out particles with area less than 6 pixels.

Particle linking was performed to generate particle trajectories and obtain binding on and off times. Biased binding events were filtered according to the procedure explained in manuscript methods, which will discard roughly half of the particle binding events. The resulting survival curves are shown in Fig S7. Without any image restoration, the particle detection and linking processes yield inaccurate binding kinetic data. Using only the 10-frame averaging image restoration, the particle tracking results yields kinetic data that is horizontally shifted. The STAWASP image restoration allows the particle detection and linking process to yield accurate data, but only when $t_{res} > 5$ frames or seconds. The high error rate before 5 frames is caused by the false particles. We therefore filter binding events shorter than $t_{cutoff} = 5$ frames to reduce the number of false particle detection errors that must be corrected manually. The close match across the data extracted from the test case movie and the actual data shows that the tracking algorithm functions sufficiently well to extract unbinding kinetic parameters. This also means that the logarithmic decay function of the influenza unbinding kinetics is not likely to be an artifact of the tracking software or the method at which we filter out biased data points.

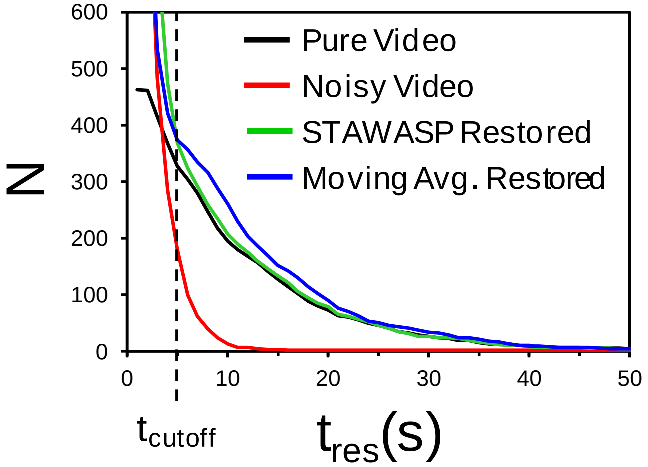

Fig S7 Comparison of binding survival curves from the SPT software before and after image restorations. Settings for the STAWASP-restored video are Nsmooth = 10, Pradius = 2, and SyncPerc = 80%. The Moving Avg-restored video uses a 10-frame moving average.

Part G. Derivation of fit equations.

We will first define Eq. 0 as the 1st-order dissociation equation

$$\frac{dN}{dt} = -k_{off} N \quad \text{Eq. 0}$$

The derivation of Eq. 3 and 5 is shown below.

1) $\ln(N/N_0) = -A \ln\left(\ln \frac{t_{res}}{\Delta t}\right) + B$ from Fig 9b log plots

2) $N/N_0 = \exp\left(-A \ln\left(\ln \frac{t_{res}}{\Delta t}\right) + B\right)$

3) $N = N_0 \exp(B) \left(\ln \frac{t_{res}}{\Delta t}\right)^{-A}$ [Eq. 3]

4) $\frac{dN}{dt} = \frac{-AN_0 \exp(B) \left(\ln \frac{t_{res}}{\Delta t}\right)^{-A-1}}{t_{res}}$

5) $\frac{dN}{dt} = -k_{off} N = -k_{off} \left[N_0 \exp(B) \left(\ln \frac{t_{res}}{\Delta t}\right)^{-A} \right]$ [Eq. 0, using N from Eq. 3]

6) $-k_{off} N_0 \exp(B) \left(\ln \frac{t_{res}}{\Delta t}\right)^{-A} = \frac{-AN_0 \exp(B) \left(\ln \frac{t_{res}}{\Delta t}\right)^{-A-1}}{t_{res}}$

7) $k_{off}(t_{res}) = \frac{A}{t_{res} \ln \frac{t_{res}}{\Delta t}}$ [Eq. 5]

The derivation of Eq. 4 and 6 is shown below.

1) $\ln\left(\frac{d(-N/N_0)}{dt_{res}}\right) = -A\ln(t_{res}) + B$ from Fig 9c log plots

2) $\frac{d(-N/N_0)}{dt_{res}} = \exp(B)t_{res}^{-A}$

3) $\int_{N_0}^{N} d(-N/N_0) = \exp(B)\int_{t_0}^{t_{res}} t_{res}^{-A} dt_{res}$

4) $N = N_0\left[1 - \frac{\exp(B)}{1-A}\left(t_{res}^{1-A} - t_0^{1-A}\right)\right]$ if A ≠ 1 [Eq. 4]

$N = N_0\left[1 - \exp(B)\ln\frac{t_{res}}{t_0}\right]$ if A = 1

5) $\frac{dN}{dt} = -k_{off}N = -k_{off}N_0\left[1 - \frac{\exp(B)}{1-A}\left(t_{res}^{1-A} - t_0^{1-A}\right)\right]$ if A ≠ 1 [Eq. 0, using N from Eq. 4]

$\frac{dN}{dt} = -k_{off}N = -k_{off}N_0\left[1 - \exp(B)\ln\frac{t_{res}}{t_0}\right]$ if A = 1

6) $k_{off}(t_{res}) = \frac{\exp(B)t_{res}^{-A}}{1 - \frac{\exp(B)}{1-A}\left(t_{res}^{1-A} - t_0^{1-A}\right)}$ if A ≠ 1 [Eq. 6]

$k_{off}(t_{res}) = \frac{\exp(B)}{t_{res}\left(1 - \exp(B)\ln\frac{t_{res}}{t_0}\right)}$ if A = 1

Part H. Confirming lipid mobility using Fluorescence Recovery after Photobleaching (FRAP).

To ensure that lipids (and glycolipids) in the SLBs are completely mobile and can thus promote multivalent binding, we conducted FRAP tests. A solution containing 0.1 mg/mL of R18 was flown into the channels containing SLBs for 4 hours. Excess R18 was rinsed away using MES buffer. A 561 nm laser with a diameter of 12 μm was focused at the bilayer to photobleach the R18 in the bilayer. The average intensity of the bleached spot was measured over 10 min, along with a reference spot located far enough away from the photobleached spot (sample FRAP images are provided elsewhere [1]). The recovery curve was normalized using the following equation:

$$f_k(t) = \frac{\left[F_k(t) - F_c(t)\right] - \left[F_k(0) - F_c(0)\right]}{\left[F_{k,PB} - F_{c,PB}\right] - \left[F_k(0) - F_c(0)\right]}$$

where $F_k(t)$ and $F_c(t)$ respectively are the average fluorescence intensities of the bleached and reference spots. $F_{k,PB}$ and $F_{c,PB}$ are the fluorescence intensities of the spots before bleaching. The bleaching of fluorophores is completed at $t = 0$. The normalized recovery curve was then fitted using the equation derived by Axelrod et al. [2], which has two fit parameters: M_f (mobile fraction) and τ_d (characteristic time of diffusion). The full equation along with associated terms are provided below.

$$f_{k,fit}(t) = M_f \left[\frac{F_{k,fit} - F_{k,fit}(0)}{F_{k,fit}(\infty) - F_{k,fit}(0)} \right]$$

$$F_{k,fit}(t) = vK^{-v}\Gamma(v)\chi(2K,2v)$$

$$v = (1 + 2t/\tau_d)^{-1}$$

K is the solution for $F_k(0) = F_{k,BP} K^{-1}(1 - \exp(-K))$

$\Gamma(v)$ is the gamma distribution

$\chi(2K,2v)$ chi-square probability distribution with $2K$ degrees of freedom at $2v$.

The diffusion coefficient can be solved for according to D = $R^2/4\tau_d$, where R is roughly 6 μm in our setup and corresponds to the laser radius at e^{-2} height. Example FRAP images are provided elsewhere[1]. A sample FRAP recovery curve is shown in Figs S8, and the diffusion coefficient (D) and fraction of lipids that are mobile (or mobile fraction, M_f) are shown in Table S1. Note that the mobile fractions and diffusion coefficients are reported for the R18 dye, not the glycolipids themselves. However, the mobility of R18 in lipids is a good indicator for the mobility of lipids and glycolipids. Dye-labeled G_{M1} has already been shown to be mobile with a diffusivity of 0.77 μm^2/s [3]. These results also suggest stationary viruses seen in our SPT videos are more likely to be held in place due to multivalent binding as opposed to binding to immobile glycolipids.

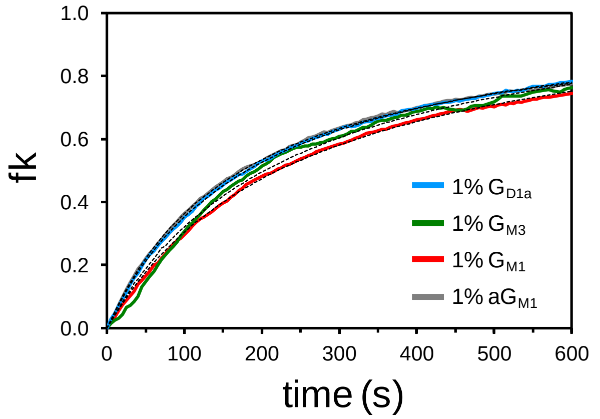

Fig S8 Example set of FRAP recovery curves. Black dotted lines are the fits for the recovery curve.

Table S1 Diffusion coefficient and mobile fractions of bilayers. R18 = octadecyl rhodamine B. Alexa-G_{M1} is a G_{M1} molecule with a fluorescent Alexa 594 probe conjugated to the sugar groups.

Bilayer	Fluorescent Probe	Diff. Coef. ($\mu m^2/s$)	Mobile Fraction
1% aG_{M1} 2% POPG 97% POPC	R18	0.40±0.12	0.97±0.06
1% G_{M1} 1% POPG 98% POPC	R18	0.31±0.06	1.04±0.03
1% G_{M3} 1% POPG 98% POPC	R18	0.28±0.01	1.07±0.04
1% G_{D1a} 0% POPG 99% POPC	R18	0.36±0.03	1.04±0.02
†1% G_{M1} 70%POPC 29% Other Lipids	Alexa594-G_{M1}	0.77	N/A

†Data from Supplemental Materials of work by L. Chao and S. Daniel [3].

References

1. Lee D, Thapar V, Clancy P, Daniel S. Stochastic fusion simulations and experiments suggest passive and active roles of hemagglutinin during membrane fusion. BIOPHYS J. 2014;106(4):843-54. doi: http://dx.doi.org/10.1016/j.bpj.2013.12.048.

2. Axelrod D, Koppel DE, Schlessinger J, Elson E, Webb WW. Mobility measurement by analysis of fluorescence photobleaching recovery kinetics. BIOPHYS J. 1976;16(9):1055-69.

3. Chao L, Daniel S. Measuring the Partitioning Kinetics of Membrane Biomolecules Using Patterned Two-Phase Coexistant Lipid Bilayers. J AM CHEM SOC. 2011;133(39):15635-43. doi: 10.1021/ja205274g.
